# The Role of Verbs in Sentence Production

**DOI:** 10.3389/fpsyg.2020.00189

**Published:** 2020-02-19

**Authors:** Inés Antón-Méndez

**Affiliations:** Discipline of Linguistics, University of New England, Armidale, NSW, Australia

**Keywords:** sentence production, syntax, verbs, hierarchical incrementality, sentence elicitation

## Abstract

To investigate the role of verbs in sentence production, the experiment reported here employed a simple sentence elicitation technique based on separate elicitor images for the different sentence constituents: subject, verb, and verbal modifier. This permitted presenting them in different temporal configurations to see whether the time taken to start uttering the subject of a sentence was contingent on having access to information about the action that would determine verb selection. The results show that sentence onset latencies varied in relation to the presentation of the verb elicitor, suggesting that sentence processing depends crucially on having access to the information pertaining to the verb. What is more, increases in the lexical frequency of the actual verbs used significantly reduced onset latencies for the subject noun as expected if the verb lemmas have to be retrieved before the sentence can be processed. Among other things, this argues against strict linearity and in favor of hierarchical incrementality in sentence production. Additionally, the results hint at the possibility that other obligatory sentence constituents [namely, direct objects (DOs) in transitive sentences] may also have to be available before the sentence can be processed.

## Introduction

Sentences are not born fully formed: they are the product of a complex process that requires first forming a conceptual representation that can be given linguistic form, then retrieving the right words related to that pre-linguistic message and putting them in the right configuration, and finally converting that bundle into a series of muscle movements that will result in the outward expression of the initial communicative intention (Levelt, 1989). This article is concerned with the initial stages of this process.

At issue is the question of whether it is possible to start preparing a sentence for production before some structurally critical sentence constituents are, at the very least, conceptually available. That is, whether a speaker can, on knowing what the topic/subject of a sentence will be in an SVO language such as English, put the machinery in motion even if the rest of the utterance is still un(der)determined. It is not hard to imagine that, in certain circumstances, the topic of the sentence may be selected before anything else is. For example, it has been found that often more salient entities tend to be encoded as subjects of sentences (Prat-Sala and Branigan, 2000; Gleitman et al., 2007; Myachykov and Tomlin, 2008), with flow-on consequences for the rest of the sentence (e.g., passive vs active voice, or alternative perspective descriptions). On the one hand, this could mean that prominence dictates which entity takes the topical function of sentence subject in English, perhaps, as Bock et al. (2004) review concludes, because this prominence correlates with predicability. In other words, speakers may tend to start sentences with entities about which something can be said: aboutees (Bock and Ferreira, 2014, p. 22). But it also suggests that speakers may be selecting the sentence’s topic (and subject) before they have made up their mind about what exactly will come after since entity prominence can affect not only what form the sentence will take (i.e., active or passive), but also the choice of verb (i.e., chase or flee; Gleitman et al., 2007).

Such a scenario is compatible with the considerable amount of evidence pointing to the incremental nature of sentence production (e.g., Meyer, 1996; Schriefers et al., 1998; van de Velde et al., 2014; Brown-Schmidt and Konopka, 2015; Zhao and Yang, 2016) as initially hypothesized by Kempen and Hoenkamp (1987). According to this, speakers do not necessarily wait for all the bits and pieces of a phrase or sentence to have been processed and set in their places before they start uttering it. Instead, speakers often appear to be able to start their utterances as soon as some of the constituents become available (see, for example, Griffin, 2001; Gleitman et al., 2007; Allum and Wheeldon, 2009), and even seamlessly add material to a linguistic unit on the fly (Brown-Schmidt and Konopka, 2008, 2015). Clearly, this favors efficiency and fluency in speech.

However, incrementality in sentence production does not necessarily imply that processing follows the sentence’s surface form. For sentences to be more or less freely assemblable on the basis of constituent availability and in a strictly linear manner, the processing of earlier constituents should not depend on requirements of later constituents—i.e., the subject could constrain verb choices but it should not itself be constrained by a verb’s specifications. But this condition does not always hold: for example, in languages with rich morpho-syntax where the specific relationships between constituents matter for the final form of the sentence (Norcliffe et al., 2015), strict linearity would be unfeasible. And even in languages with more impoverished morpho-syntax such as English, there are dependencies between different sentence constituents that would be expected to limit the extent to which some parts of a sentence can be processed separately from other later coming parts. That is, there are underlying structural relationship between different constituents in a sentence that can only be properly established once all the constituents in question are available to the speaker. This may explain why much of the evidence for linear incrementality concerns optional constituents such as adjectives and prepositional modifiers of a noun (Brown-Schmidt and Konopka, 2008, 2015; Zhao and Yang, 2016), while research on full sentences has tended to support a more hierarchical incrementality where the processing of certain constituents does seem to depend on the processing of later ones with which they are structurally related (J. Lee and Thompson, 2011; Momma et al., 2016, 2018; Kidd et al., 2018).

Hierarchical incrementality presupposes that speakers cannot start processing a sentence on the basis of a single constituent, but instead need to have at least a rough idea of the overall relationships between the constituents within that sentence (E. K. Lee et al., 2013). A strong version of the hierarchical incrementality account would require access to specific lexical items such as the verb to enable building the initial sentence outline (as in the model proposed by Bock and Levelt, 1994) since some structural relationships are based on individual word specifications (e.g., nothing in the semantics of “donate” and “give” explains why the receiver can be an object for the latter but not the former: “the gentleman gave/^∗^donated the library his books”). That would be lemma-driven scaffolding. A weaker version, structure-driven scaffolding, proposes that the basic hierarchical structure could be derived from the more abstract conceptual representation of the communicative intention, before specific lexical items are retrieved (Griffin and Bock, 2000; Bock and Ferreira, 2014). Both possibilities are compatible with the finding that, when describing a picturable event, speakers appear to use the first few hundred milliseconds, around 300–400, scanning the whole scene before settling on the component of the scene that would be produced first (Griffin and Bock, 2000). It is also possible that both lemma-driven and structure-driven scaffolding operate during sentence production (Bock and Ferreira, 2014)—syntactic processing is not monolithic, and it may allow for different circumstances favoring the engagement of different mechanisms.

Given the central role verbs play in sentences, one straightforward prediction that can be derived from hierarchical incrementality is that either the verb or the conceptual representation underlying it (which will be referred to as the “action” henceforth^1^) should be essential for initiating sentence production (E. K. Lee et al., 2013). That is, it should not be possible to start preparing a sentence before the action is known since it is what ties the different participants in an event together and what will ultimately, in the form of a verb, underpin the relationships between the different sentence constituents. In fact, early psycholinguistic accounts of sentence production assumed verbs played a critical role in the generation of a sentence by being responsible for its basic structural shape (Levelt, 1989; Bock and Levelt, 1994; Ferreira, 2000)—an assumption that has found some empirical support from, among others, eye tracking and priming experiments (Pickering and Branigan, 1998; Melinger and Dobel, 2005; Hwang and Kaiser, 2014; Antón-Méndez, 2017; Sauppe, 2017). For example, Antón-Méndez (2017, Figure 3) found facilitation during picture description when the action in the sentence to be produced had been linguistically primed, but not when the subject had been visually primed, suggesting the verb plays a major role in sentence planning. And Hwang and Kaiser (2014) found that the action region was fixated before the subject region during sentence production in English. In sum, there are reasons to believe that the action/verb is processed early during sentence production.

However, not all published evidence points to verbs being necessary to start uttering a sentence. Schriefers et al. (1998) found a semantic interference effect on the verb when the sentence was Verb-Subject (in German), but not when it was Subject-Verb, which implies processing of the verb was not interfering with processing of the subject when the latter was uttered first. Additionally, while some authors have found that the verb’s internal arguments [i.e., direct objects (DOs) and subjects of unaccusative verbs] depend on verb retrieval for their processing (J. Lee and Thompson, 2011; Momma et al., 2016, 2017, 2018), some of this research also finds that typical subjects (i.e., those in transitive and unergative sentences) do not. In particular, Momma et al. (2016, 2018). have found that the time it takes to start uttering a sentence increases when there is semantic interference affecting the verb, but this only happens for sentences where the DO precedes the verb (in Japanese; Momma et al., 2016), or when the subject is semantically more related to a DO than to a typical subject (i.e., for English unaccusatives; Momma et al., 2018)^2^, but not when the first constituent in the sentence is a typical sentence subject. This difference between the reliance on verb access of the two types of verbal arguments could be explained by the closer relationship between verbs and their internal arguments (i.e., DOs) than between verbs an their external arguments (i.e., subjects of accusatives and unergatives; Kratzer, 1996), but it puts into question the notion that verbs are always necessary for overall sentence processing.

Another reason to believe subjects may have a preeminent role in sentence production is the clear preference shown in language comprehension to interpret the first encountered argument as the sentence’s subject even in languages, like German, where syntactic function is morphologically marked and even in cases where there is a preference for DOs to appear first (Bornkessel and Schlesewsky, 2006). Not only has this subject-first strategy been found in a number of spoken languages (see Bornkessel and Schlesewsky, 2006, for a review), but also on at least one signed one (Krebs et al., 2018) attesting to its universality. While a strategy that applies in comprehension does not necessarily have to have a place in production (after all, the two processes are associated with different starting points and different aims; see, e.g., Pozzan and Antón-Méndez, 2017), it is also true that this apparent propensity in comprehension to assume the first argument mentioned must be the subject may be a reflection of a more fundamental role of this argument in language in general.

Efforts to test the relevance of verbs in sentence production have relied on indirect evidence: priming (e.g., Antón-Méndez, 2017), semantic interference (e.g., Momma et al., 2016), distributed looks to event participants (e.g., Sauppe, 2017; Konopka, 2019), etc. The reason is that the sorts of sentence elicitation techniques used in the past, based on descriptions of pictured events, conflate the actions with the actors making it difficult to manipulate them independently—that is, in the eliciting pictures, the information that would ultimately be conveyed in the form of a verb is not isolatable from the depiction of the actors in the event. In contrast, the experiment reported below attempts to address the question of the role of verbs in sentence production in a more direct way by looking at whether a sentence could start to be processed in a linear fashion on the basis of the handle provided by just having an aboutee that will become the intended sentence’s subject, even when the action to be described is not yet known. This is achieved by means of a new sentence elicitation technique based on separate elicitors for the different sentence parts. This technique allows for the different elicitors to be presented to the speaker in different sequential orders which, in turn, makes it possible to study whether starting to utter a sentence’s subject is contingent on having access to the action expressed by the verb. The underlying rationale is that the timing of the constituent that minimally lets the speaker start preparing the sentence will determine how long it takes for the sentence to be uttered. Having access to what will become the subject of a sentence is clearly necessary as this is the first constituent to be uttered in SVO sentences like those targeted here. But the crucial point is whether it is also sufficient for the speaker to start processing the sentence. If it is, the presentation of a subject-eliciting picture should allow the speaker to access the associated lemma, prepare the noun phrase (NP), and assign it the role of subject to which additional sentence constituents will be added later. In that case, sentence onset times should be consistent with the presentation point of the subject elicitor. On the other hand, if access to the subject elicitor is not sufficient for the speaker to ready the sentence structure in which it has to be inserted, sentence onset times will not be consistent with the timing of the subject elicitor but should instead depend on the timing of the constituent that satisfies the minimum requirements for the sentence structure to be computed. In particular, if verbs are strictly necessary for sentence processing, sentence onset latencies should be longer when the verb elicitors appear after than when they appear before the subject elicitors.

## Materials and Methods

### Participants

Thirty-two native speakers of English (34% males) participated in this experiment in exchange for a compensation of AU$7. Their mean age was 26.3 y.o. (*SD* = 8.3). Participants were residents of Armidale (Australia) and most were students at the University of New England. There were 6 participants in lists 1 and 2, and 5 in lists 3–6 (see section “Design” below).

### Materials

**FIGURE 1:**
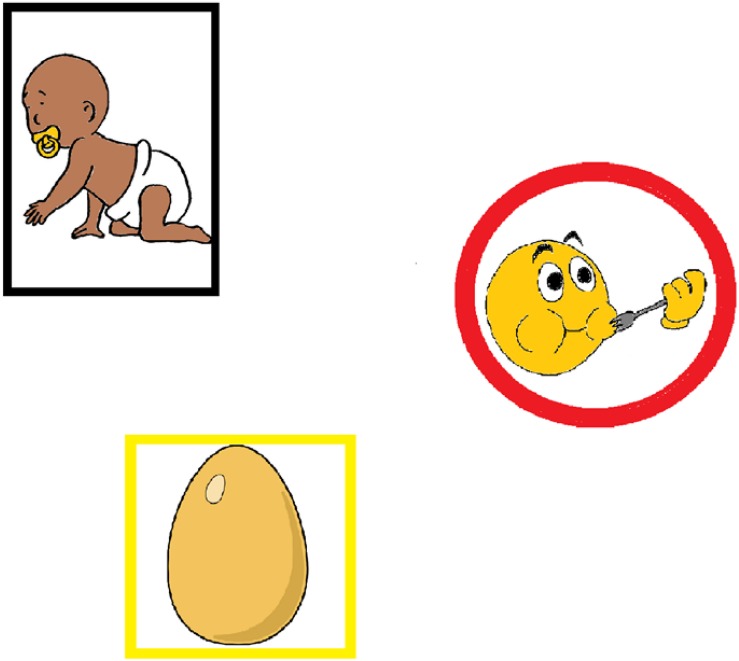
Stimuli presentation. Images were presented in one of six possible points along an imaginary circumference. In this instance, they are occupying positions 2, 4, and 6.

Stimuli consisted of colored drawings depicting either a person (e.g., a baby, *N* = 36); a thing or animal (e.g., an egg, *N* = 36, only two were animals), or an action (e.g., eating, *N* = 36). The images were sourced from picture databases (Snodgrass and Vanderwart, 1980; Masterson and Druks, 1998) and the internet, and modified as necessary by the author. To increase the distinctiveness of the different kinds of elicitor images, the persons were framed by a black rectangle; the things were framed by a yellow square, and the actions were framed by a red circle (see Figure 1). This was necessary in order to ensure participants did not automatically assign the function of subject to the image that was presented first, as may otherwise have happened given the evidence toward a subject-first strategy (see section “Introduction”).

The elicitor images were presented in sets of three, one of each kind. These image triplets represented a proposition that could be expressed as a single simple sentence: persons would elicit concepts most naturally encoded as sentence subjects, actions would elicit concepts to be encoded as verbs, and things/animals should be encoded either as DOs or as prepositional modifiers to the verb depending on verb type (e.g., “the baby ate *an egg*” or “the woman ran *down the street*”). Within a triplet, the images appeared on screen in a staged manner with intervals of 250 ms between them. The order of presentation was counterbalanced across six lists (see section “Design” below for further details).

Different types of verbs were included: transitive verbs, which require the presence of both a subject and a DO; and intransitive verbs, which only require the presence of a subject. Given the empirical evidence on different types of intransitives being processed differently (Agnew et al., 2014; Meltzer-Asscher et al., 2015; Momma et al., 2018), intransitive verbs were of two types: unergatives and unaccusatives. These differ in their semantic, and possibly also syntactic, relation with their subjects (see footnote 1). There were 12 verbs of each of the three types: transitive, unergative, and unaccusative.

The intended target names and example sentences can be found in the Supplementary Appendix.

### Design

The staged presentation resulted in six different presentation orders for each image triplet, namely: Person first, Action second, and Thing last; Person/Thing/Action; Action/Person/Thing; Action/Thing/Person; Thing/Person/Action; and Thing/Action/Person. The first elicitor image was accompanied by a click which served to align the recording with the visual presentation. The different presentation orders were counterbalanced across six item lists.

The interval between the onset of each of the three images in a triplet was 250 ms. This interval was long enough for lexical access to occur before the next image was presented (175–250 ms; Indefrey and Levelt, 2004; Costa et al., 2009) but short enough to allow fluent speech even if the sentence was produced in a strictly linear manner (eye-voice span to fluenly insert a modifier in an NP is approximately 500 ms in Brown-Schmidt and Konopka, 2008; eye-voice span to onset of subject NP is approximately 500 ms in Griffin and Bock, 2000; furthermore, the average time span between subject noun onset and main verb onset in this experiment was 642 ms, exceeding the 500 ms maximum possible time lapse between the first and last images), and also short enough that the staging was not found obtrusive or distracting—indeed, during debriefing, participants acknowledged having noticed something but assumed it was unintended upload delays or screen flickering.

To prevent participants anticipating where the elicitor for a particular sentence constituent would appear and developing a strategy, images could appear in any of six different positions derived from dividing an imaginary circumference of approximately 16 cm diameter into six arcs of equal length with the single restriction being that two images were never presented in adjacent positions. Each triplet of images was assigned one of the 12 possible display configurations, and each configuration appeared an equal number of times in each list and condition. For example, the baby always appeared in position 5, eating in position 1, and the egg in position 3. However, in each of the six lists, the order in which the three positions were filled, namely, the presentation order, was different.

Although there are six possible presentation orders giving rise to six different lists, for the purposes of addressing the predictions (see below), the experimental design was actually concerned with the presentation of either the Action or the Thing relative to the Person. In consequence, the presentation orders were collapsed into two independent variables: Action-Relative and Thing-Relative Presentation conditions, each with two levels: Action or Thing before Person, and Action or Thing after Person. Additionally, the experimental design included a third independent variable, Verb Type, with three levels: transitive, unergative, and unaccusative verbs.

In sum, the current study responds to a 2 × 2 × 3 factorial design with repeated measures: two Action-Relative Presentations, two Thing-Relative Presentations, and three Verb Types.

### Procedure

Participants were first familiarized with the images. These were presented one by one for the participant to name, blocked by elicitor kind—first the Persons, then the Actions, and finally the Things. If the participant was unsure about what the image represented or used a label that was not appropriate (e.g., “happy” instead of “smile”), the experimenter provided the correct term. Otherwise, participants were free to use any label that was compatible with the image and valid for the purpose of the experiment, even if it was not the originally intended name. Stimulus presentation was self-paced.

The reason participants were free to choose the images’ labels was to prevent their trying to remember what word the researcher wanted them to use, thus ensuring subsequent sentence production was as spontaneous and fluent as possible. The percentage of produced sentences containing words other than the intended targets was 28.5. The alternatives used by the participants were mostly common (near) synonyms of the intended target (e.g., ocean for sea, kitten for cat, skip for jump, etc.) or other labels that could apply to the image (e.g., businessman for man, doctor for dentist, etc.; see the Appendix).

In a second phase, participants saw the image triplets consisting of one Person, one Action, and one Thing (see Figure 1 and the Appendix), and they were asked to make simple sentences with them. The images should have been intuitively associated, respectively, with a sentence’s subject, verb, and verbal complement or modifier but, furthermore, participants were explicitly instructed to describe what was happening such that “the person was doing something.” Four practice items helped participants get used to the task. Their verbal responses were recorded.

The experiment was delivered using the experimental software E-Prime (version 2.0, Psychology Software Tools, 2012) on a Dell Latitude E6430 laptop. The whole session lasted 13 min on average and was audio recorded in its entirety with a portable digital recorder (Edirol by Roland R-09HR).

### Data Processing

The produced sentences were first transcribed and coded in terms of their validity and their fluency. Regarding validity, sentences were considered valid if they conformed to expectations, i.e., they were of the form subject-verb-DO/prepositional modifier, and all the images had been labeled appropriately, i.e., either as intended or in a way compatible with the experimental design (see the Appendix for accepted alternative labels used by participants). Any departure from the expected sentence form (e.g., “the sun dries the swimmer” instead of “the swimmer dries in the sun”), addition of material in the form of comments or elaboration, self-corrections while uttering the sentence, and delays due to physiological processes (e.g., throat clearing, laughing) rendered a sentence invalid and resulted in its exclusion from the analyses. Sentences were also considered invalid if the verb had been replaced by one of a different type (e.g., “smell” in its transitive form instead of “stink,” “read” instead of “speak”).

Regarding fluency, sentences were considered fluent if there were no internal pauses (greater than approximately 200 ms), and disfluent otherwise. Disfluent sentences were excluded from the analyses (*N* = 225, 24% of valid responses; they were evenly distributed across the different Presentation conditions).

Onset latencies were measured manually using ELAN software (version 5.2, Max Planck Institute for Psycholinguistics, 2018). Latencies measured the time elapsed between the onset of the Person image and the onset of the noun (N) in the subject NP, that is, the start of the subject N in relation to when it could conceivably start to be processed. Latencies that differed by more than 3 s.d. from the mean of their Presentation condition and Verb Type were excluded from the analyses reported below.

### Predictions

The predictions of this experiment concern the effect of different Presentation conditions on onset latencies for the subject N relative to when the elicitor of the sentence’s subject, the Person, appeared on screen.

If knowing the action is necessary to start processing the sentence, it should not be possible to process the subject NP until the verb elicitor, the Action, has been made available. That would mean subject N onset latencies following the presentation of the Person would be slower in conditions where the Action followed than when it preceded the Person.

Otherwise, if the subject can be prepared independently of the predicate, the subject NP could be processed as soon as the subject elicitor is available. That would mean subject N onset latencies once the Person appears on screen should be equally fast for all Presentation conditions regardless of whether elicitors for other sentence constituents had already been presented. However, this might not apply to unaccusative sentences given the different relation between the subject and the verb in these sentences—even if typical subjects could be processed independently of the verb, subjects that are originally internal arguments of the verb may show dependence on the verb. In particular, if subjects can in principle be processed before the verb is known, subjects of unaccusatives should be treated just like other subjects in conditions where the Person preceded the Action. However, they may undergo a different, more involved processing in the converse Presentation condition.

In sum, conditions where the subject elicitor precedes the verb elicitor should result in *slower* subject N onset latencies (relative to opportunity to produce said subject) if the subject cannot be prepared independently of the verb/action. If having a potential sentence subject is sufficient to start processing the sentence, whether the verb elicitor is available or not would be immaterial, and there should be no difference in subject N onset latencies between conditions in which the verb elicitor preceded and those in which it followed the subject elicitor (at least for transitive and unergative sentences).

Additionally, since the Things accompanying intransitive verbs should be encoded as optional verbal modifiers (e.g., “the farmer shouted *at the cat*”), the incremental nature of sentence production should ensure that the presentation position of the Thing should not affect subject N onset times. In contrast, there is evidence that internal and obligatory arguments of a verb cannot be processed independently of it (Momma et al., 2016, 2018), which could impact the behavior in the case of transitive sentences. However, it should be borne in mind that the predictions in the current study are primarily concerned with the production of the subject in relation to the verb rather than the production of the DO. Looking at it from that perspective, it may be that the verb itself is sufficient to build a basic blueprint of the sentence including a place holder for a DO in such a way that a speaker would not need to wait until the actual elicitor for the DO has been processed in order to start uttering the sentence or to complete it without disfluencies. Alternatively, if the verb cannot be processed until its internal arguments are known, and the subject cannot be processed until the verb has been processed, subject N onset latencies should be longer when the object elicitor follows than when it precedes the subject elicitor for transitive sentences only.

## Results

Of a total of 1152 utterances, there were 940 valid sentences (81.60%; 672 fully on-target sentences, and 268 sentences with accepted substitutions), and 212 invalid ones (18.40%). Of the 940 valid sentences, 715 were considered fluent (76.06%). The total numbers of responses falling under each coding category according to condition can be found in Table 1.

**TABLE 1:** Number of responses in each coding category for the different Presentation conditions and Verb Types.

**Category**	**On-target**	**Acceptable**	**Invalid**	**Valid disfluent**
**Person/Action**				
Transitive	130	38	23	28
Unergative	89	65	37	42
Unaccusative	115	32	45	44
**Action/Person**				
Transitive	147	31	14	36
Unergative	84	66	42	39
Unaccusative	107	34	51	35

Totals	672	266	212	224

**Person/Thing**				
Transitive	133	40	19	33
Unergative	84	68	40	43
Unaccusative	115	30	47	39
**Thing/Person**				
Transitive	144	29	19	31
Unergative	89	63	40	38
Unaccusative	107	36	49	40

**FIGURE 2:**
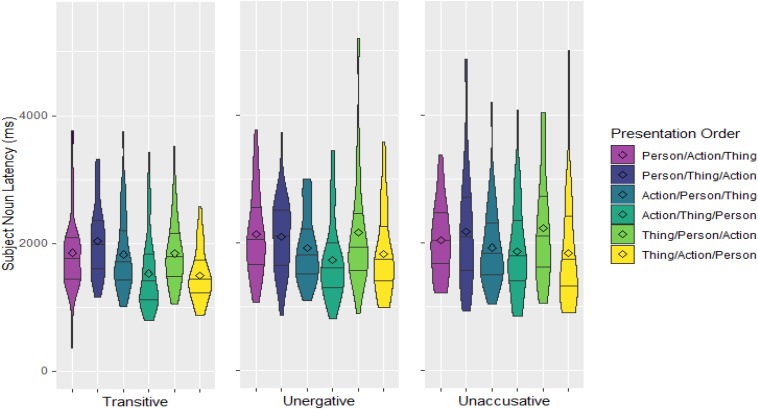
Densities and quartiles of subject noun latencies in ms relative to onset of the Person image for all presentation orders and Verb Types. The length of the shapes reflects the spread of latencies and the width is an indication of the number of observations at any particular latency point. The lines divide the data into the longest 25% onset latencies, the second longest 25% onset latencies, etc. The diamond shapes denote the mean onset latencies of each group.

**FIGURE 3:**
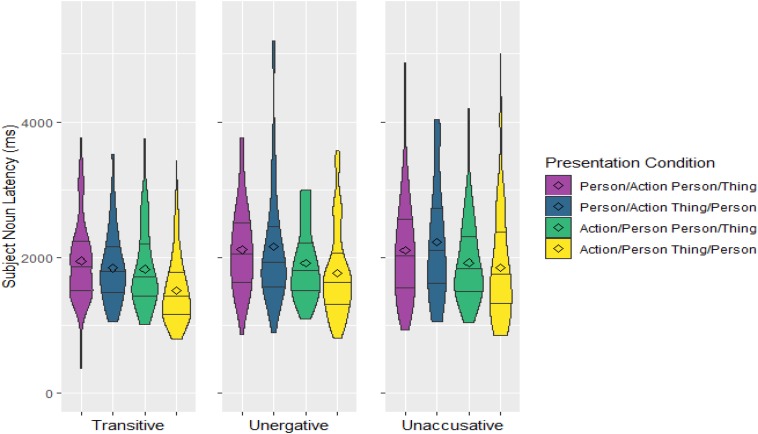
Densities and quartiles of subject noun latencies in ms relative to onset of the Person image for the three Verb Types and the four Presentation conditions of interest: two based on presentation order of the Person and the Action, and two based on presentation order of the Person and the Thing. The length of the shapes reflects the spread of latencies and the width is an indication of the number of observations at any particular latency point. The lines divide the data into the longest 25% onset latencies, the second longest 25% onset latencies, etc. The diamond shapes denote the mean onset latencies of each group.

Average onset latencies for uttering the subject N relative to the time the Person appeared on screen for each presentation order and Verb Type can be seen in Figure 2 (excluding outliers). As already explained above, to address the predictions, which are predicated on the appearance of the Person with respect to either the Action *or* the Thing, the six presentation orders were collapsed according to when the Person appeared (1) relative to the Actions (Action-Relative Presentation) and (2) relative to the Things (Thing-Relative Presentation; see Figure 3).

Analyses were carried out with the statistical program R (Version 3.3.1, R Core Team, 2015) using the lme4 package (Bates et al., 2015). The main analysis consisted of a linear mixed-effects (LME) model on the logarithmic transformation of subject N onset latencies (e.g., “baby” in a sentence such as “the baby is eating the egg”) relative to presentation onset of the Person. The model included the fixed effects of, and interactions between, Action-Relative Presentation (contrast coded; Person/Action = −0.5; Action/Person = 0.5), Thing-Relative Presentation (contrast coded; Person/Thing = −0.5; Thing/Person = 0.5), and Verb Type. Helmert coding was used for the three-level variable of Verb Type, which was analyzed in two parts: first the two intransitive verbs were contrasted against each other, and then they were contrasted together against the transitive verbs (Piccinini, 2016). The data included only fluent^3^ valid sentences, and excluded three outliers (latencies greater than 3 s.d. from the mean of each Presentation condition and Verb Type; they comprised 0.4% of data points). A maximal random effect structure was attempted first but had to be simplified following a backward best-path procedure to overcome convergence issues (Barr et al., 2013). This resulted in a model with only the random intercepts. A summary of the model’s results is given in Table 2.

**TABLE 2:** LME model and results.

Log latency Sbj N relative to Person onset ∼ 1 + Action-Relative Presentation * Thing- Relative Presentation * Verb Type + (1 | Subject) + (1 | Item)			
**Factor**	**Estimate (s)**	***SE***	***p***
Intercept	0.61	0.04	< 0.001***
Action-Relative Presentation	–0.14	0.02	< 0.001***
Thing-Relative Presentation	–0.08	0.02	< 0.001***
Verb Type: Unergatives vs Unaccusatives	0.01	0.04	0.850
Verb Type: Transitives vs Intransitives	–0.12	0.04	0.007**
Action-Relative Presentation* Thing-Relative Presentation	–0.10	0.04	0.014*
Action-Relative Presentation *Verb Type (Ug/Uc)	0.02	0.05	0.710
Action-Relative Presentation *Verb Type (T/I)	0.00	0.06	0.952
Thing-Relative Presentation *Verb Type (Ug/Uc)	0.02	0.05	0.571
Thing-Relative Presentation *Verb Type (T/I)	–0.10	0.06	0.062.
Action-Rel Present*Thing-Rel Present *Verb Type (Ug/Uc)	0.02	0.11	0.848
Action-Rel Present*Thing-Rel Present*Verb Type (T/I)	–0.07	0.11	0.514

**Note:* Ug = unergative; Uc = unaccusative; T = transitive; I = intransitive.*

The results indicate that, regarding the Action-Relative Presentation, the subject N takes less time to be uttered once the Person appears when the Action was already on screen than when it was not; and, regarding the Thing-Relative Presentation, it was also uttered faster when the Thing was already on screen than when it was not. The interaction of these two variables was also significant as the difference between the two Thing-Relative Presentation conditions is smaller than that between the two Action-Relative Presentation conditions: while the delay in the Person/Action relative to the Action/Person condition averages 294 ms, the delay in the Person/Thing relative to the Thing/Person condition only averages 180 ms, less than the onset asynchrony between images. Additionally, subject N latencies were longer for intransitive sentences than transitive ones; but not different between unergatives and unaccusatives. There was a marginal interaction between Thing-Relative Presentation and Verb Type in terms of transitivity (Thing-Relative Presentation^∗^Verb Type (T/I) in Table 2), and this reflects the fact that the difference between the two Thing-Relative Presentation conditions was larger for the transitive sentences than for the two intransitive ones. In fact, it may just be this larger difference between the two Thing-Relative Presentation conditions for transitive verbs what drives the significant difference of the main effect of Thing-Relative Presentation as well as its interaction with Action-Relative Presentation (see Figure 3). Indeed, separate *post hoc* analyses on the transitive and intransitive verbs point in this direction: while, for transitives, both the main effect of Thing-Relative Presentation and the interaction between the two Presentation variables are significant (Thing-Relative Presentation: *estimate* = −0.13, *SE* = 0.03, *p* < 0.001; Interaction: *estimate* = −0.16, *SE* = 0.06, *p* = 0.014); for intransitives, the main effect of Thing-Relative Presentation is only marginally significant and the interaction is not significant (Thing-Relative Presentation: *estimate* = −0.05, *SE* = 0.03, *p* = 0.059; Interaction: *estimate* = −0.07, *SE* = 0.05, *p* = 0.172).

One potential problem with the current design is that the presentation timing of the critical image, the Person, varies with condition. Although the relation with the other two images is variable in all cases, it means that whenever the Person was presented first, the Action would have always followed it and, whenever the Person was presented last, the Action would have always preceded it. If there is any advantage in terms of processing for images presented last, this could have contributed to the effect of Action-Relative Presentation. To see whether the effect can be replicated when the position of the Person is held constant, an additional LME was conducted on the two conditions with the Person appearing second: Action/Person/Thing vs Thing/Person/Action. Since the results of the full model hint to a Thing-Relative Presentation effect for transitive sentences which has the potential to obscure the effect in point, i.e., that of Action-Relative Presentation, only the data for the intransitive verbs was included in this analysis (*N* = 146). The log-transformed onset latencies for the subject N were modeled with the fixed factor of condition (Action/Person/Thing and Thing/Person/Action) and random intercepts over subjects and items (as more elaborate random factor structures failed to converge). The main effect of condition was significant (*estimate* = −0.09, *SE* = 0.04, *p* = 0.030), confirming that the effect found in the main analysis is not due to the presentation position of the Person: the subject N was reliably produced earlier when the Action had been presented before the Person^4^.

**FIGURE 4:**
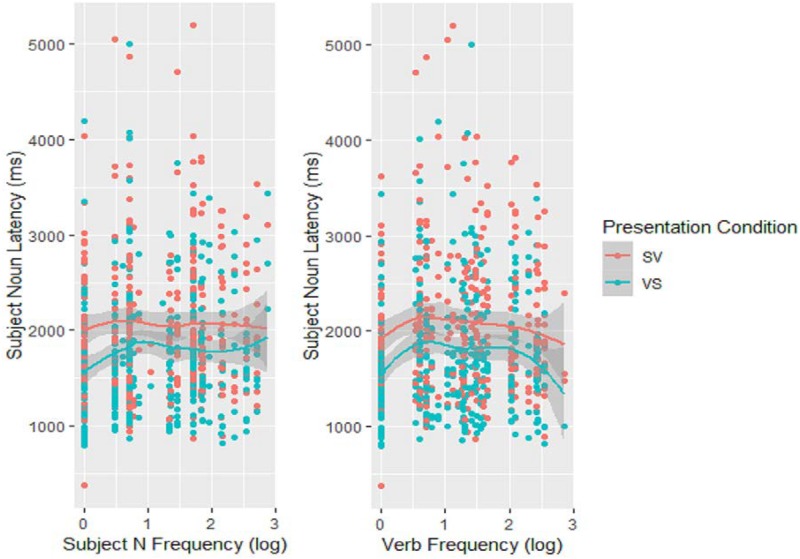
Scatter plots of the effect of either the subject noun **(left)** or the verb **(right)** log frequency on subject noun latencies, with Loess lines fitted.

In principle, the effect of the Action image on subject onset latencies could result either from processing the depicted action at the conceptual level or from accessing the verb’s lemma. To distinguish between the two, I looked at the dependence of onset latencies on a property specific to lexical access: lexical frequency. The log frequencies of the actual labels used by participants (either subject Ns or verbs) were included in an LME model on log transformed subject N onset latencies which also included the Action-Relative Presentation variable as a fixed factor and, due to convergence issues, only the intercepts over the random factors of subject and item. Both subject N and verb frequencies affected onset times significantly (Subject N: *estimate* = 0.13, *SE* = 0.04, *p* < 0.001; Verb: *estimate* = 0.10, *SE* = 0.04, *p* = 0.013) but in opposite directions: higher verb frequencies tended to reduce latencies, while higher subject N frequencies appeared to increase them (see Figure 4). This was also reflected in a significant interaction of the two frequency effects (*estimate* = −0.09, *SE* = 0.02, *p* < 0.001). On the other hand, neither of the two frequency effects interacted with the Presentation condition (both *p*’s > 0.168). These results indicate that starting to utter the subject N depends on having accessed the specific verb that will appear in the final utterance although, unexpectedly, they also show that this is the case whether the verb is the limiting factor (Person/Action conditions) or not (Action/Person conditions). On the other hand, the inverse effect of the subject N’s frequency is hard to explain.

## Discussion

In this experiment, participants produced simple sentences on the basis of three images depicting: a person to elicit a subject, an action to elicit a verb, and a thing to elicit a DO or verbal modifier. The three elicitor images appeared one after the other at intervals of 250 ms, and in different orders. Under these conditions, it would have been possible timewise for a speaker to simply start to prepare the NP that would become the subject of the target sentence at the point at which the subject elicitor appeared on screen, then continue adding constituents to the sentence as they became available, and still produce a correct fluent sentence. Such performance would have pointed toward fairly strict linear incrementality and support accounts of sentence production that do not require knowing what the action is in order to start preparing the sentence. But this is not what happened. Instead, speakers took 294 ms more on average to utter the sentence when the verb elicitor was not present at the time the subject elicitor was made available (Person/Action conditions) than when it was (Action/Person conditions). When the verb elicitor was already available, speakers started uttering a fluent sentence on average about 1760 ms after the subject elicitor appeared—an interval similar to that found in other event description experiments (Griffin and Bock, 2000; Sauppe et al., 2013; Antón-Méndez, 2017; Kidd et al., 2018). The difference of almost 300 ms found when the subject elicitor appeared before the verb elicitor coincides with the average presentation asynchrony between Person and Action images in the Person/Action condition which, for all the sentences included in the analysis, was 329 ms on average (since the asynchrony was 250 ms in some trials and 500 ms in others, depending on whether the thing image intervened between the two). This is compatible with a scenario in which the speaker, after seeing the Person, waited for the Action to appear before preparing the sentence—that is, it seems as if speakers could only process the subject NP after the concept underlying the verb (the action) was known. As such, these results add to the empirical evidence provided by eye-tracking measures that English speakers tend to first fixate on the action region of a picture (Hwang and Kaiser, 2014). Furthermore, the fact that starting to utter the sentence was significantly affected by the log frequency of the verb such that more frequent verb lemmas resulted in shorter latencies strongly suggests that speakers were not only waiting to start processing the sentence until the underlying concept for the action was known, but also until the particular verb to be used in the sentence was accessed. In contrast, and as expected from the incremental nature of sentence production, it did not seem to be the case that speakers waited until all the elicitors needed for to the to-be-uttered sentence were available (i.e., until all the images were on screen) in order to start preparing the sentence. While speakers did take longer to start uttering the sentence when the object/modifier elicitor was presented after the subject elicitor, the average difference was only 180 ms—far shorter than the average stimulus asynchrony between the Person and the Thing images in the Person/Thing condition, which was 337 ms. The longer latencies for the Person/Thing condition seem to instead be the result of the speaker waiting for the object elicitor to appear in the case of transitive sentences, since it is only for these that the Person/Thing condition was reliably associated with longer latencies than the Thing/Person condition when the verb elicitor was already available (Action/Person condition; see Figure 3)—that is, having access to the verb elicitor appears to be insufficient to start processing a transitive sentence. The evidence for the intransitive sentences does not show the same specific dependency of sentence processing on the availability of the object elicitor.

These results quite unambiguously show the importance in sentence planning of, at minimum, the verb’s conceptual representation but, most likely, the verb lemma itself. They help explain the event apprehension phase identified in several eye-tracing picture description experiments. The first few hundred milliseconds after a depicted event is presented appear to be used by speakers to do a global inspection of the scene before the eyes are directed to the part of it that would be produced first (e.g., Griffin and Bock, 2000; Antón-Méndez, 2017). We do not know what exactly is happening during this apprehension phase but, given these results, it is likely that what speakers are doing is trying to identify the action associated with the event and even select the verb that would best describe it so that the event participants can then be allotted to the right syntactic functions. In previous experimental designs, the “event” included an action that was most of the times only deducible from assessing the relations between the event participants (e.g., a copper was chasing a robber instead of just running because there was a robber appearing to be fleeing) and/or was conflated with one of the participants (e.g., a person kicking a ball, where the “kicking” is part of the “person”). Thus, if speakers need to first know the action in order to start preparing the sentence, this could easily take the form of a global scene inspection. In short, the initial phase of event apprehension may simply be the speakers’ way of figuring out what action is being depicted to then retrieve the verb that is needed to plan the sentence (see also Konopka, 2019).

Early access of specific verb lemmas in sentence production makes sense given that languages have some degree of arbitrariness, that a sentence’s morphosyntax expresses the relations (both conceptual and formal) between constituents, and that the verb is at the core of the relations between the major, obligatory constituents. Therefore, it is reasonable to suppose that building a sentence’s syntactic frame depends not just on knowing the action at a conceptual level, but also on knowing the specific verb that would be used. For example, the syntactic frame associated with a verb such as “search” would be different than that for a verb such as “look for,” even though they are synonymous at the conceptual level; and the argument assignment for a verb such as “frighten” would be different than that for a verb such as “fear,” even though, again, they can be considered conceptually synonymous (i.e., someone is finding something scary: “something frightens someone” vs “someone fears something”). Thus, particular verbs’ specifications are needed to produce correct sentences at least some of the time. And, in fact, the interval associated with event apprehension, from 0 to approximately 400 ms (Griffin and Bock, 2000) can amply accommodate lexical access (175–250 ms; Indefrey and Levelt, 2004; Costa et al., 2009), so selecting the specific verb that would best describe the identified action could potentially also be part of the event apprehension phase.

In any case, the results of the present experiment firmly show that knowledge of an event’s action at a conceptual level is needed to arrive at a representation that suffices to start processing a sentence, and that uttering the subject is likely to depend on access to the verb lemma. These results align with theories that assume sentence production proceeds in a hierarchical fashion and, moreover, on the basis of specific verbs (Levelt, 1989; Bock and Levelt, 1994; Ferreira, 2000).

While the present results allow us to conclude that the action is more pre-eminent in sentence production than the conceptual underpinning of a verbal modifier in intransitive sentences, as predicted by the models of sentence production just cited, they do not allow us to say whether the action has a more privileged role than the concept underlying the subject. It could be that *all* the obligatory components of a sentence have to be available for the basic structural scaffolding of the sentence to be built, as hinted by the fact that, in transitive sentences, onset times were also delayed when the object elicitor was presented last. Research in a verb-first language would be needed to establish whether the subject has the same status as the verb in terms of sentence production.

These results failed to replicate previous findings suggesting that typical subjects do not depend on verb retrieval for their processing (Schriefers et al., 1998; Momma et al., 2016, 2018). It is not clear what is responsible for the discrepancy but one possibility is that it has to do with the methodology. Unlike the current design, the studies cited used the picture-word interference task based on semantic interference. One potential problem is the arguable unreliability of semantic interference for verbs (Schnur et al., 2002). Another potential problem is the fact that semantic interference is very time-sensitive and it could be that, for example, distractors missed the point at which they could interfere with the structural planning initiated by the verb for unergatives but not for unaccusatives (Momma et al., 2018), since the latter are generally associated with longer latencies. More research will be needed to identify the source of the discrepancy.

## Conclusion

The results presented here show that sentence planning depends on knowledge of the action and the verb expressing it. This argues against strictly linear accounts of sentence production and in favor of the strong form of hierarchical incrementality whereby sentence planning relies on accessing the verb lemma. The results also indicate that other obligatory sentence constituents (namely, DOs in transitive sentences) may be equally needed to commence sentence processing. If so, the same may be expected of subjects. By allowing researchers to manipulate sentence constituents independently, the new methodology introduced in this paper could be used to further research into these issues and help us elucidate what is going on in a speaker’s mind when she is preparing a sentence.

## Data Availability Statement

The datasets generated for this study are available on request to the corresponding author.

## Ethics Statement

The studies involving human participants were reviewed and approved by University of New England Human Ethics Committee. The patients/participants provided their written informed consent to participate in this study.

## Author Contributions

The author confirms being the sole contributor of this work and has approved it for publication.

## Conflict of Interest

The author declares that the research was conducted in the absence of any commercial or financial relationships that could be construed as a potential conflict of interest.

## Footnotes

1 This is not meant to imply that all verbs express actions. The term is just a convenient shortcut because, in this research, most of the verbs did indeed refer to actions.

2 Briefly, subjects of unergative verbs are typically agents, i.e., they initiate and are responsible for the action expressed by the verb; while subjects of unaccusative verbs do not (e.g., Sancho would not intentionally have started the action when “Sancho fell from his donkey”). Therefore, subjects of unaccusative verbs have been postulated to start life as internal arguments of the verb—that is, they are initially treated like typical direct objects, rather than typical subjects (Burzio, 1986).

3 As already mentioned, it would be perfectly possible to produce a fluent sentence without waiting for the next picture to appear since the average time span between subject noun onset and main verb onset was 642 ms, exceeding the 500 ms maximum possible time lapse between the first and last images.

4 In the interest of thoroughness, an analysis including the transitive sentences was also conducted. The effect of condition was also significant in this analysis (*estimate* = −0.07, *SE* = 0.03, *p* = 0.044).
