# Supplementary material for: The Role of Verbs in Sentence Production
Source: Front Psychol. 2020 Feb 19;11:189. doi: 10.3389/fpsyg.2020.00189 (PMC7042407; doi:10.3389/fpsyg.2020.00189)
Supplement: Supplementary file 1 [file Data_Sheet_1.pdf]

Appendix: Intended image names (in bold) and accepted alternatives used by participants (below the intended names), together with example sentences (the most common forms) that could be made with the three words.

	Verb Type	People	Actions	Things/Animals
	Pract-Trans	**chauffeur**	**lock**	**trunk**
		**The chauffeur locked the trunk**		
	Pract-Unerg	**carpenter**	**spit**	**bucket**
		**The carpenter spat in the bucket**		
	Pract-Unerg	**shepherd**	**pray**	**church**
		**The shepherd prayed in church**		
	Pract-Unacc	**fireman**	**flip**	**back**
		**The fireman flipped on his back**		
1	Transitive	**secretary**	**cut**	**note**
		receptionist	saw	letter, list
		**The secretary cut the note**		
2	Transitive	**nurse**	**cook**	**fish**
		**The nurse cooked some fish**		
3	Transitive	**clown**	**comb**	**wig**
			brush	hair, mannequin
		**The clown combed his wig**		
4	Transitive	**girl**	**draw**	**car**
		woman		
		**The girl drew a car**		
5	Transitive	**sailor**	**sew**	**sock**
		soldier	stitch, darn, knit	
		**The sailor sewed a sock**		
6	Transitive	**nun**	**water**	**bush**
		nurse		garden
		**The nun watered the bush**		
7	Transitive	**chef**	**peel**	**onion**
		baker, cook		
		**The chef peeled the onion**		
8	Transitive	**tailor**	**iron**	**shirt**
		man		clothes
		**The tailor ironed a shirt**		

9	Transitive	**policeman**	**type**	**list**
		police officer	write	checklist, report, letter, notes
		**The policeman typed a list**		
10	Transitive	**baby**	**eat**	**egg**
		**The baby ate an egg**		
11	Transitive	**pirate**	**knit**	**scarf**
		sailor	saw	
		**The pirate knitted a scarf**		
12	Transitive	**dentist**	**dust**	**cupboard**
		doctor	clean, brush	cabinet, dresser, drawers
		**The dentist dusted the cupboard**		
13	Unaccusative	**doctor**	**fall**	**floor**
		physician	slip	ground, pavement, path
		**The doctor fell on the floor**		
14	Unaccusative	**bride**	**faint**	**pillow**
		woman		
		**The bride fainted on the pillow**		
15	Unaccusative	**king**	**hang**	**branch**
			fall	
		**The king hung from a branch**		
16	Unaccusative	**witch**	**drown**	**pool**
				swimming pool
		**The witch drowned in the pool**		
17	Unaccusative	**soldier**	**slip**	**banana**
		sailor		banana peel, banana skin
		**The soldier slipped on a banana**		
18	Unaccusative	**cowboy**	**stink**	**skunk**
		man	smell	
		**The cowboy stank like a skunk**		
19	Unaccusative	**swimmer**	**dry**	**sun**
		person, man		
		**The swimmer dried in the sun**		
20	Unaccusative	**singer**	**shine**	**stage**
		dancer, performer		

		**The singer shone on stage**		
21	Unaccusative	**surfer**	**float**	**river**
				stream, creek, ravine
		**The surfer floated down the river**		
22	Unaccusative	**gardener**	**slide**	**ravine**
		man	slip	
		**The gardener slided down the ravine**		
23	Unaccusative	**artist**	**sleep**	**mattress**
		painter		bed
		**The artist slept on the mattress**		
24	Unaccusative	**detective**	**roll**	**stairs**
		**The detective rolled down the stairs**		
25	Unergative	**woman**	**run**	**street**
		lady, girl	walk	city
		**The woman ran down the street**		
26	Unergative	**teacher**	**sit**	**table**
				desk
		**The teacher sat on the table**		
27	Unergative	**man**	**talk**	**book**
		businessman	speak	
		**The man talked about the book**		
28	Unergative	**queen**	**swim**	**sea**
				ocean, beach, pool
		**The queen swam in the sea**		
29	Unergative	**astronaut**	**jump**	**hoop**
			skip	
		**The astronaut jumped through the hoop**		
30	Unergative	**maid**	**dance**	**broom**
				broomstick
		**The maid danced with the broom**		
31	Unergative	**boy**	**laugh**	**house**
				home
		**The boy laughed at the house**		

32	Unergative	**mechanic**	**smile**	**dog**
				puppy
		**The mechanic smiled at the dog**		
33	Unergative	**driver**	**cry**	**graveyard**
				cemetery, gravestone, grave
		**The driver cried at the graveyard**		
34	Unergative	**judge**	**walk**	**train**
		lawyer	run	train station
		**the judge walked to the train**		
35	Unergative	**baker**	**kneel**	**pew**
		chef	pray	church, bench, chair
		**The baker kneeled at the pew**		
36	Unergative	**farmer**	**shout**	**cat**
			yell, scream, call	kitten
		**The farmer shouted at the cat**		
